# Development of a Remote Online Collaborative Medical School Pathology Curriculum with Clinical Correlations, across Several International Sites, through the Covid-19 Pandemic

**DOI:** 10.1007/s40670-021-01212-2

**Published:** 2021-01-20

**Authors:** Gerardo E. Guiter, Sandra Sapia, Alexander I. Wright, Gordon G. A. Hutchins, Thurayya Arayssi

**Affiliations:** 1 Division of Medical Education, Weill Cornell Medicine-Qatar, 445 East 69 Street, RM 432, New York, NY 10021 USA; 2 Division of Medical Education, Weill Cornell Medicine- Qatar, Qatar Foundation - Education City, P.O. Box 24144, Doha, Qatar; 3 Section of Pathology, Leeds Institute of Medical Research, University of Leeds, 4.11 Wellcome Trust Brenner Building, St James’s University Hospital, Beckett Street, Leeds, LS9 7TF UK; 4 Leeds Teaching Hospitals NHS Trust/University of Leeds. Histopathology and Molecular Pathology, St James’ University Hospital, Beckett Street, Leeds, LS9 7TF UK

**Keywords:** Pathology, Online, Remote, Curriculum, Medical, School

## Abstract

**Introduction:**

Due to the Covid-19 social distancing restrictions, in March 2020, Weill Cornell Medicine-Qatar decided to replace students’ clinical instruction with novel online electives. Hence, we implemented an innovative online and remote pathology curriculum, anchored on virtual microscopy and Zoom videoconferencing: ideal tools to support online teaching.

**Objective:**

To assess a new curriculum implementation at Weill Cornell Medicine-Qatar.

**Materials and Methods:**

This for-credit, 2-week elective included 6 synchronous Zoom sessions where complex clinicopathological cases were discussed in small groups. We used open access digital microscopy slides from the University of Leeds’ Virtual Pathology Library (http://www.virtualpathology.leeds.ac.uk/slides/library/). Students independently prepared for these sessions by reviewing cases, slides, readings, and questions in advance (asynchronous self-directed learning anchored on a flipped classroom model), and wrote a final review of a case. An assessment and feedback were given to each student.

**Results:**

Four elective iterations were offered to a total of 29 students, with learners and faculty spread over 4 countries. During the Zoom sessions, students controlled the digital slides and offered their own diagnoses, followed by group discussions to strengthen autonomy and confidence. We surveyed learners about the elective’s performance (program evaluation). Students conveyed high levels of satisfaction about the elective’s overall quality, their pathology learning and online interactions, with minimal challenges related to the remote nature of the course.

**Discussion and Conclusions:**

Technological innovations mitigate sudden disruptions in medical education. A remote curriculum allows instruction *at any distance, at any time, from anywhere,* enhancing educational exchanges, flexibility and globalization in medical education.

## Introduction

Weill Cornell Medicine-Qatar (WCM-Q) is an international site of Weill Cornell Medicine. Established in 2001 as a partnership between Cornell University and Qatar Foundation, WCM-Q is part of Cornell University in Ithaca, New York, and shares the tripartite mission of Weill Cornell Medicine-New York (WCM-NY): a dedication to excellence in education, patient care, and research. It offers an integrated program of pre-medical and medical studies leading to the Cornell University MD degree.

WCM-Q medical school curriculum is guided by key principles of enhancing the integration between the foundational sciences and clinical learning, and introducing students to patient care from the first day of their studies. The curriculum is divided into 3 phases. During Phase 1, which extends over 18 months, students complete their foundational sciences courses. Phase 2 is dedicated to 12 months of clinical clerkships at primary and tertiary care academic centers in Doha, Qatar. Phase 3 focuses on advanced clinical experiences and the completion of a scholarly capstone project.

Due to the global Covid-19 pandemic, in March 2020, WCM-Q suspended all clinical instruction in a move aligned with the Association of American Medical Colleges (AAMC) and the Liaison Committee on Medical Education (LCME) recommendations [1–3] and similar decisions by other educational institutions in North America, Asia, and Europe [4–7]. This approach to protecting students was sensible particularly in light of past experiences, such as when 16 medical students exposed to severe acute respiratory syndrome (SARS) patients in a hospital during the Hong Kong 2003 outbreak unfortunately contracted the disease [8].

As a result, our institution faced a sudden challenge: to adapt to the new situation and enable our students to progress in the curriculum safely. Consequently, WCM-Q decided to replace our learners’ clinical work with a portfolio of novel electives to be delivered entirely online starting in mid-April 2020, covering various fields of expertise. The strategy included offering 2- or 4-week electives to provide students with enhanced flexibility if they needed to return to the clinical space on short notice.

At that challenging time, the authors of this project believed that pathology teaching offered a superb solution for our institutional needs, based on the use of virtual microscopy and digital slides. These are ideally suited for delivering an undergraduate pathology curriculum [9], and many institutions in North America and Europe have already switched to this approach [10–12]. Indeed, WCM-Q has been using virtual slides for pathology and histology instruction throughout all basic science courses since 2005, and the American Board of Pathology certification exam began using virtual microscopy in 2003 [13]. Large academic medical centers have ample experience with virtual microscopy and employing digital slides for undergraduate teaching [14] and clinical diagnostic purposes [15], and most recently, during the Covid-19 pandemic [16, 17]. In the US, a corresponding relaxation of government regulations has allowed pathologists to review and report pathology specimens from remote, non-Clinical Laboratory Improvement Amendments (CLIA) certified facilities [17], expanding the use of digital slides with remote reviews.

However, virtual microscopy and digital slides in medical school education have traditionally accompanied face-to-face “in-situ” interactions, with very few remote applications and mostly targeting post-graduate training [18, 19]. To communicate with our learners within the social distancing restrictions imposed by the Covid-19 pandemic, our project sought to develop and implement an undergraduate innovative pathology collaborative learning experience. Students and instructors would connect remotely and share––synchronously and asynchronously––virtual microscopy, digital slides, and other images, as well as discussions, questions, and answers through the Zoom videoconferencing system, currently available at WCM-Q as an institutional subscription. We targeted WCM-Q medical students that had already completed their foundational sciences courses. The theoretical framework for this new curriculum was anchored on the flipped classroom model, with students initially learning and constructing new knowledge on their own, and faculty subsequently utilizing online classroom time for problem solving, formative teaching, and answering students’ questions.

## Materials and Methods

### Design of the Elective

We designed a 2-week online elective with the overarching goal that learners, by focusing on the study of several malignant tumors and their precursor lesions, would acquire an enhanced knowledge of malignant diseases and therefore the basis for robust understandings of oncologic pathology and oncology. The elective was available for any student who had successfully completed our foundational sciences curriculum. Students passing the elective would gain appropriate credit as determined by institutional policy.

The elective included a total of 6 interactive, synchronous live sessions using Zoom videoconferencing (3 sessions per week). We decided to accept a maximum of 10 students per elective iteration, believing that an instructional approach based on small groups best suited our program, supporting increased student participation and positive learning outcomes.

Two WCM-Q and Department of Pathology and Laboratory Medicine WCM-NY faculty members developed 9 clinicopathological cases which included links to access remotely corresponding digital slides from the University of Leeds Virtual Pathology Slide Library (http://www.virtualpathology.leeds.ac.uk/slides/library/). This open access collection of slides is digitized and curated by trained pathologists and stored with anonymized clinical information, used for teaching and training. Slides can be viewed on standard computer screens and mobile devices and are globally accessible. All cases included an initial clinical vignette describing a patient presentation and diagnostic imaging and laboratory results, followed by biopsy and/or surgical excision histopathological findings. The cases also presented illustrations of gross specimens, immunohistochemical images, and for some, descriptions and results of advanced molecular studies. The cases focused on uterine cervical, colonic, pulmonary, and mammary oncologic pathology, and included a list of differential diagnoses, with the University of Leeds Slide Library virtual images illustrating alternative but meaningful and related pathological entities.

As our foundational sciences curriculum at WCM-Q includes general and systemic pathology, our students had already acquired a basic knowledge of this discipline. We accordingly created cases that were somewhat more complex than those our learners had already studied, so they would continue incorporating new information and constructing new understandings in pathology throughout this elective.

We included in all cases 2 types of questions aimed at topics addressed during the sessions: first order basic queries and more complex United Stated Medical Licensing Examination (USMLE) type items. Students were expected to answer these questions to the best of their knowledge drawn from readings and general preparation. We included the USMLE items with the following 3 objectives: first and most importantly, to ensure students had learned their materials and increased their knowledge; second, to strengthen their self-directed learning (SDL) and subsequent self-assessment skills; and third, to contribute to their preparation for the USMLE Step 1, an exam taken by most of our learners prior to graduation.

We decided to interactively teach and connect with the students via the Zoom videoconferencing platform. Zoom allows for screen sharing and control at a distance by any participant––a significant plus, we believed, that could enhance learner autonomy and self-discovery skills by granting students full remote control to point out specific areas while describing virtual pathology or radiology images. In addition, we also chose to make use of the built-in Zoom polling as another way to increase student contributions during case discussions.

As we planned the elective, we decided that students would be expected to independently prepare for these Zoom sessions by reviewing the cases and digital slides, completing assigned readings, and answering all basic and USMLE-type questions in advance (asynchronous SDL). Accordingly, students were provided with access to all materials, including the virtual pathology slides, 72 h before each Zoom meeting. This independent preparation was factored into the students’ assessment (please see below). Therefore, in order to support our students’ success, the program comprised two components of approximately equal importance: a preparatory asynchronous SDL step followed by synchronous interactive sessions with live Zoom discussions.

The elective was hosted on the WCM-Q Canvas Learning Management System (LMS). Each iteration included a unique centralized site on Canvas, allowing for faculty and learner interactions, student submissions, access to teaching files, images, and other documents, and assessment of students after completion of the elective.

Students were assessed through direct faculty observations of their preparation for case discussions, in class participation and contributions, responses to basic and USMLE-type questions, and also by writing a final 500-word summary review of a case discussed during the elective. This review had to address all significant pathological and related clinical issues. It was open-ended, and no written rubric was provided to the students; instead, they were given verbal instructions at the beginning and end of the elective. The weight of this assignment was 50% of the overall final grade.

A final written assessment of each student’s performance included several items graded on a 5-point Likert type rating scale, including formative and summative comments. In addition, the faculty provided oral formative feedback to the students through one-on-one Zoom meetings. The final course grade was assigned as Pass/Fail.

This project was submitted to the WCM-Q Institutional Review Board (IRB) and determined IRB exempt. In addition, the institutional leadership was fully supportive of our initiative, quickly approving the elective for credit recognition and ensuring all the resources were readily accessible to the teaching faculty.

## Results

### Implementation of the Elective

Four elective iterations were offered to a total of 29 students between April 19 and June 25. The first iteration included 10 students, the second included 5 students, and the third and fourth iterations included 7 students each. Learners and faculty were spread over 4 different countries (Qatar, Saudi Arabia, Peru and Argentina).

**Fig. 1:**
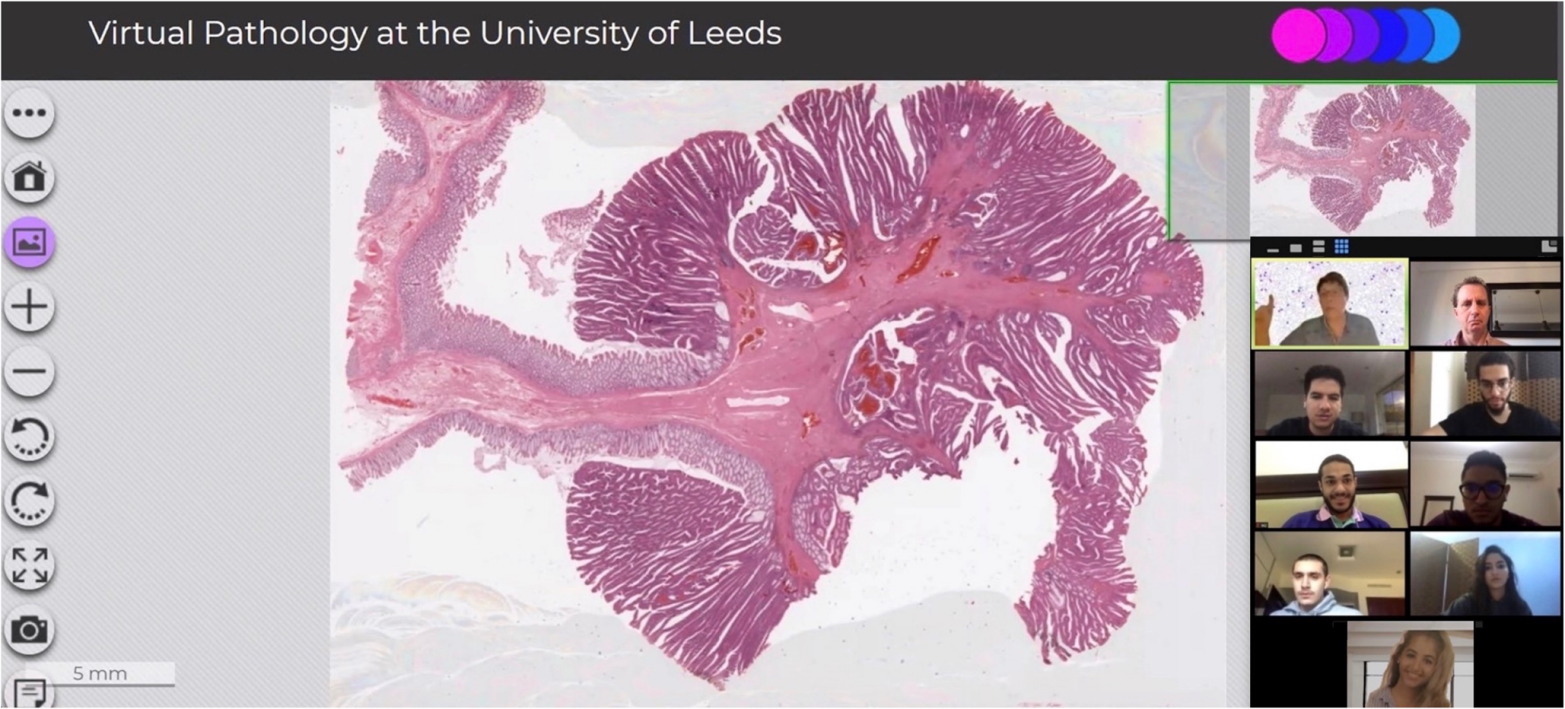
WCM-Q faculty and students discussing a virtual microscopy pathology slide via Zoom videoconferencing

The 2 participating WCM-Q/WCM-NY faculty facilitated the Zoom instruction. Following the program design, we met with students 3 times per week for sessions lasting 90 to 120 min. While discussing cases, the students had many opportunities to take control of the virtual slides, moving the images and zooming in and out at their discretion, and selecting areas of interest to share with the group. They also described their initial preliminary pathological diagnoses based on their evaluation of the virtual slides. In so doing, the learners “acted” as pathologists in control of their cases, similar to what happens at pathology departments during consensus meetings where difficult cases are shown to colleagues. In general, learners took turns to offer their preliminary and differential diagnoses, with faculty facilitating active participation and contributions by each student. In addition, towards the end of each case discussion, students answered the basic and USMLE-type questions anonymously, through the Zoom polling. Subsequently, responses were analyzed by the group, in order to enrich the discussions. This questions and answers section also allowed faculty to assess learners’ prior preparatory SDL effectiveness, identify any knowledge gaps, make all necessary corrections, and provide formative feedback (Fig. 1).

We did not experience any difficulties with the Zoom platform, which worked very well during all sessions, and encountered no “Zoombombing” [19].

### Assessment of the Elective and Students’ Feedback

We surveyed our students to acquire some preliminary impressions and data about the elective’s performance and functioning (program assessment), and to make any necessary changes. We aimed to evaluate the elective’s overall quality and the quality of student and faculty online interactions, our students’ online and remote learning of pathology, any challenges associated with their work and communications, and the most/least effective aspects of their remote learning experiences. We acknowledged that this appraisal would only be based on our learners’ input, assessment, and feedback.

A 2-part, anonymous survey, prepared by the WCM-Q Office of Educational Development (OED) for all virtual elective courses, was distributed to the 29 students who had completed our pathology elective, and 23 (79.31%) responded. Tables 1 and 2 summarize the survey results.

**Table 1:** Survey of students about the elective’s performance (program assessment)-Part A

	Mean^a^	Standard Deviation
Elective’s overall quality	4.9	0.34
Clarity of elective’s goals and objectives	4.9	0.34
Elective’s organization (syllabus, schedule, learning arrangements, support mechanisms)	4.8	0.41
Elective’s contribution to the students’ learning and understanding of oncologic pathology	4.8	0.51

^a^Calculated using a 5-point rating scale, with “1” representing the lowest possible score and “5” representing the highest possible score

**Table 2:** Survey of students about the elective’s performance (program assessment)-Part B

	Mean^a^	Standard Deviation
After completing this elective, my knowledge of some complex pathological entities and diseases has improved	4.6	0.49
After completing this elective, my recognition of the critical importance of robust clinicopathological correlations has improved	4.6	0.49
After completing this elective, my comprehension of the rationale supporting targeted therapies and preventive approaches in malignancies has improved	4.4	0.65
The elective’s Zoom sessions encouraged interactions between students	4.8	0.39
The elective’s Zoom sessions encouraged interactions between faculty and students	4.7	0.44

^a^Calculated using a 5-point Likert scale: [SD] Strongly Disagree = 1; [D] Disagree = 2; [N] Neutral = 3; [A] Agree = 4; [SA] Strongly Agree = 5

We additionally asked the students if they had or had not experienced any challenges due to their remote participation in this course. Only 1 out of 23 (4.35%) respondents answered that they had, noting “some lag in the communications.”

Finally, we asked the students to describe the most and the least effective aspects of their remote learning experiences. Virtual pathology slides, ability to communicate with peers and engage more in the activities through online learning rather than when it is face-to-face, easier communication with the professors, more relaxed environments at home, enhanced time management with no time lost in transportation to/from the college, lack of distraction by peers, solving the cases prior to the sessions and inclusion of USMLE-type questions were all reported as highly effective. Indeed, some students mentioned they preferred remote instruction over face-to-face teaching. No comments were received on the least effective aspects of the course.

During the four iterations of the course, we encountered loss of Internet during 1 of 24 sessions (4.16%), which affected the connectivity of a few students. This session was re-scheduled for the next day, resolving the problem. We also encountered loss of the virtual microscopy website connectivity during 2 of 24 sessions (8.33%). This issue, though not ideal, was solved by sharing static images (JPGs) instead, prepared in advance from the same corresponding digital slides. We did not encounter any other barriers or challenges to our remote online program.

While technical troubles, as described above, were rare, they underscored the critical importance of developing Internet and server back-up systems for institutions that offer online courses. In addition, although during our elective we used standard home-type Internet, networks that offer adequate bandwidth are crucial. In summary, efforts must be made to mitigate technical risks that can diminish student satisfaction and participation. Every student passed the elective.

## Discussion

This project involved the development and implementation of a novel undergraduate pathology curriculum at WCM-Q, built on the use of virtual microscopy, with learners and faculty interacting remotely by Zoom videoconferencing––a notably original approach in pathology teaching in medical school education.

Fortunately, we were able to design and implement this elective swiftly, as our appraisal of existing infrastructure confirmed that all the resources needed were already available. These included Zoom videoconferencing, Canvas, Information Technology assistance, secretarial support, OED collaboration, and the University of Leeds virtual slides. In addition, we obtained crucial WCM-Q leadership support, as evidenced by their quick approval of the elective for credit recognition and by ensuring that all the resources were readily accessible.

During our needs assessment, we also considered the characteristics of our targeted learners. First, they are tech savvy, critical in this elective, which is heavily reliant on technology. Second, they are familiar with flipped classrooms and therefore were well prepared for our elective entailing many aspects of the flipped classroom model. Third, our students have strong pathology foundations and are familiar with clinicopathological cases and virtual slides, since pathology teaching during the foundational courses at WCM-Q is grounded on these tools. Finally, we did not encounter any barriers to this elective. All of these factors mentioned above are critical for the implementation and success of a new medical school curriculum.

The program was very well received by the students. Based on their written and oral feedback, the remote nature of the course presented minimal challenges. Learners reported high satisfaction with the opportunities to interact online with peers and faculty, and with their learning of pathology and related topics. Indeed, some students mentioned they preferred this mode of teaching over face-to-face instruction. Reasons for this predilection included, among others, as mentioned before, enhanced time management and a lack of distraction by peers, as sometimes happens during instruction at the college. In addition, we as faculty did not encounter any difficulties with the remote delivery of our curriculum.

Our program was a response to the sudden need to interact with students at a distance. We switched from a traditional teaching paradigm of face-to-face interactions with our learners to a new ideal approach of 100% remote exchanges. This experience parallels similar needs and responses in the clinical arena. For example, in the US as a result of this pandemic, hospital pathologists have been allowed to temporarily switch reporting of cases to remote locations, following guidance by the College of American Pathologists (CAP) and the Centers for Medicare and Medicaid Services (CMS) [20–22]. In fact, as per CMS guidance, a temporary testing site may even include the pathologist’s home.

We were also interested in whether students carried on developing their SDL and self-discovery skills through this program. They were accordingly provided with cases and access to virtual slides, readings, and other activities 72 h in advance of Zoom meetings. During their preparatory time, the learners reviewed these items on their own and were expected to create differential diagnoses, eventually formulating their own specific cancer diagnosis. Thus, this preparatory time was a great opportunity for SDL and self-discovery in line with LCME standards for accreditation of medical education programs [23] and World Federation for Medical Education (WFME) basic medical education global standards for quality improvement [24]. Nevertheless, we would need a considerably extended follow-up and further appraisals to determine if this approach enhances the SDL of our students in the long-term. The same applies to including USMLE-type questions in the cases, and correlating this strategy with the USMLE performance of our learners.

During the Zoom meetings, as each student took their turn to discuss a case, they were first asked to share with the group the diagnoses they had generated during their preparatory time. This was followed by a group discussion and a final consensus diagnosis under faculty guidance. Students remotely controlled the virtual images during the discussions, selecting and showing to others via Zoom, their fields of interest on the digital slides and important tumor features to support their diagnoses. This learning strategy can strengthen student autonomy and confidence, and eventually, self-efficacy and motivation for learning. Faculty also offered formative feedback and reinforcements. In addition, sharing images, ideas, and questions and answers live through Zoom enhanced collaborative work and learning. In fact, sometimes students offered different perspectives on a difficult case, encouraging rich discussions during our meetings.

## Conclusions and Future Directions

Our experience demonstrates how enhanced flexibility and technological innovations can now effectively relieve and mitigate sudden disruptions in undergraduate medical education in the foundational sciences due to a viral pandemic, or for any other cause.

This is not the first time medical education has been affected by an infectious outbreak. The 2003 Hong Kong SARS and 2015 South Korea Middle East Respiratory Syndrome (MERS) outbreaks mandated barring students from patient contact, canceling clerkships, and closing medical schools [25–27]. Luckily, although the COVID-19 pandemic has harshly impacted education at many levels, we now have instructional tools previously unavailable. Still, it is imperative to learn from recent events, since the possibility exists of future analogous challenges. The current pandemic has encouraged instructional innovations that must be included in our medical education armamentarium.

A benefit of this remote curriculum is that it allows instruction *at any distance, for any reason, at any time, and from anywhere*. This supports flexibility and *globalization in medical education.* As virtual teaching becomes more generalized, it will allow students to interact with peers and faculty at other institutions worldwide, supporting and increasing educational exchanges.

Remote education, however, cannot replace face-to face instruction. We favor a blended system, including distanced and face-to-face teaching. Moreover, some key aspects of medical education (e.g., bedside teaching), can only occur in the context of direct contact with patients.

It is also important that policymakers and governing bodies ensure that medical schools have all the necessary resources to face the next “emergency” in undergraduate medical education. This includes technology and faculty development. Similarly, it would be useful for appropriate national (e.g., LCME in the US) and supranational (e.g., WFME) organizations to establish some common rules, standards, and guidelines for medical education at a distance so that high-quality remote instruction is assured and maintained.

Lastly, our work should be replicated by others and evaluated accordingly. Future research should look at hard evidence of learning outcomes associated with remote online instruction.

## Data Availability

all data and materials support the published claims and comply with field standards. All data generated or analyzed during this study are included in this article.
